# Myeloperoxidase can differentiate between sepsis and non-infectious SIRS and predicts mortality in intensive care patients with SIRS

**DOI:** 10.1186/s40635-017-0157-y

**Published:** 2017-09-15

**Authors:** Irene T. Schrijver, Hans Kemperman, Mark Roest, Jozef Kesecioglu, Dylan W. de Lange

**Affiliations:** 1 Department of Intensive Care Medicine, University Medical Centre, University of Utrecht, Heidelberglaan 100, 3584 CX Utrecht, The Netherlands; 2 Department of Clinical Chemistry and Haematology, University Medical Centre, University of Utrecht, Heidelberglaan 100, Utrecht, 3584 CX The Netherlands; 3 Synapse B.V, Maastricht, The Netherlands

**Keywords:** Myeloperoxidase, Sepsis, Critical care, Biomarkers, Mortality, SIRS

## Abstract

**Background:**

Systemic inflammatory response syndrome (SIRS) is a clinical syndrome following inflammation. Clinically, it is difficult to distinguish SIRS following an infection, i.e., sepsis, from non-infectious SIRS. Myeloperoxidase is a hemeprotein stored in the neutrophil azurophilic granules and is one of the main pillars of neutrophil attack. Therefore, we hypothesized that myeloperoxidase can differentiate between sepsis and non-infectious SIRS in patients with systemic inflammatory response syndrome in the intensive care unit (ICU).

**Methods:**

An observational single-center cohort study was conducted measuring myeloperoxidase in patients with SIRS in the first 48 h after admission. The outcomes were established using predefined definitions. Thirty-day mortality was retrospectively assessed.

**Results:**

We found significantly higher levels of myeloperoxidase in patients with sepsis and septic shock compared to patients without sepsis (60 ng/ml versus 43 ng/ml, *P* = 0.002). Myeloperoxidase levels were related to 30-day mortality (*P* = 0.032), and high MPO levels on top of a high APACHE IV score further increased mortality risk.

**Conclusions:**

We show that myeloperoxidase is a potentially novel biomarker for sepsis in the ICU. Myeloperoxidase could eventually help in diagnosing sepsis and predicting mortality. However, more research is necessary to confirm our results.

## Background

Systemic inflammatory response syndrome (SIRS), a clinical syndrome following inflammation, is characterized by tachycardia, tachypnea, fever, and leukocytosis [1]. In the intensive care unit (ICU), over 50% of patients show symptoms of SIRS during their hospital stay [2]. SIRS can be attributed to an infection (which is called “sepsis”) or to a non-infectious inflammatory stimulus, like polytrauma, surgery, pancreatitis, or burns. Both non-infectious SIRS and sepsis often result in hemodynamic shock, acute kidney failure, and organ dysfunction [3]. It is vital to discriminate between non-infectious SIRS and sepsis since timely treatment with antibiotics is lifesaving in case of sepsis [4]. Clinically, it is hard to distinguish non-infectious SIRS from sepsis, and current diagnostic tools like microbiologic cultures take time and have low sensitivity [5]. Additional diagnostic tools are needed to differentiate between SIRS with and without infection [6].

Myeloperoxidase (MPO) is a hemeprotein stored in the neutrophil azurophilic granules. MPO synthesizes hypochlorous acid and other reactive oxidants to phagocytize ingested bacteria. Thus, MPO is an important component of innate immunity and one of the main pillars in the neutrophils attack of bacteria [7, 8]. Neutrophils are the first cell type to react in the host immune defense. Activated neutrophils spill their granule contents, including MPO, into plasma. Therefore, MPO could be a diagnostic biomarker differentiating between SIRS without infection and sepsis. Secondly, we hypothesize that MPO could be predictive for mortality. When found in plasma, MPO and the reactive oxygen species formed by MPO will damage native cells, which could result in higher mortality. Thus, MPO could be a marker for the collateral damage caused by neutrophils when repelling invading pathogens.

We conducted a prospective study in a cohort of SIRS patients in the ICU to determine the discriminative value of MPO between non-infective SIRS and sepsis and the prognostic value of MPO as a biomarker for mortality.

## Methods

### Patient population

The study consists of patients, older than 18 years of age, with two or more SIRS criteria at admission to the ICU, and an anticipated ICU stay of more than 24 h, that were included over a 6-month period [9]. The SIRS criteria used were temperature (> 38.0 °C or < 36.0 °C), leukocyte count (< 4 × 10^9^ L^−1^ or > 12 × 10^9^ L^−1^), tachypnea (respiratory rate > 20 per minute or PCO_2_ < 4.3 kPa), and tachycardia (heart frequency > 90 beats per minute) [10].

### Blood collection and measurement of MPO

In the first 48 h following ICU admission, blood was sampled twice and collected in heparinized tubes with an estimated median plasma volume of 700 μl. The levels of MPO were measured using a semi-automated ELISA on a TECAN Freedom EVO robot (Tecan, Männedorf, Switzerland). For the myeloperoxidase-ELISA, three different antibodies were used: R&D Rat anti-human MPO (2 μg/ml) as a coating antibody and R&D Goat anti-human MPO, biotin-labeled (0.2 μg/ml) as a second and DAKO Streptavidin, and HRP-coupled (0.71 μg/ml) as a third antibody. Relative light units from HRP activity were measured using Supersignal West Pico Chemiluminescent substrate (Thermo Scientific, Waltham, MA, USA) by Spectramax L luminescence microplate reader (MDS Analytical Technologies, Concord, Canada). C-reactive protein (CRP) and leukocytes were measured in the same sample. The maximum value of the two samples was the variable used.

### Definitions

The outcomes (no sepsis, sepsis, and septic shock) were defined as the consensus definitions by Singer et al., determined within 24 h after admission [11]. In short, sepsis was defined as suspected infection plus signs of organ failure (defined as at least two points on the Sequential Organ Failure Assessment (SOFA) score). Septic shock was defined as sepsis with persistence of systolic hypotension despite adequate fluid resuscitation necessitating vasopressor use. The Acute Physiologic and Chronic Health Evaluation IV score (APACHE IV predicted mortality) was determined in the first 24 h. Mortality was retrospectively assessed by searching the hospital database.

### Statistical analyses

The variable used for the MPO variable was the maximum value of MPO of the two blood samples. All variables were assessed by the Shapiro-Wilk and Kolmogorov-Smirnov’s d statistic on normality. Baseline characteristic comparisons were made using Mann-Whitney U, Chi square or Kruskall-Wallis tests for skewed variables, and students *T* test or Chi square for normally distributed variables. The relation of MPO with no sepsis, sepsis, and septic shock was assessed using the Kruskall-Wallis and the Mann-Whitney U test. Statistical analyses were performed using SPSS 21.0 (SPSS Inc., Armonk, NY).

The patients were divided in quintiles based on their MPO levels. The relationship between the MPO quintiles with mortality was determined using the Spearman’s rho statistic. Next, the population was divided into four groups based on the MPO levels and APACHE IV score (Table 2). The cutoffs of MPO and APACHE IV were made between the fourth and fifth quintile. Cumulative survival was calculated by applying the Kaplan-Meier method. Statistical differences between survival curves were analyzed using Cox-proportional hazards regression analysis.

A 2-tailed *P* < 0.05 was considered statistically significant.

## Results

### Baseline characteristics

We included 313 patients with SIRS with a median age of 61 years [interquartile range (IQR) 48–72]. Patients were predominantly male (62%), and 26% of the patients were admitted with suspicion of infection, and 24% died within 30 days after inclusions. During the first 10 days of admission, 36% of patients had an infection. Of our 313 patients, three patients had missing values for MPO and they were excluded for further analyses. We were unable to calculate the SOFA score of six patients and the APACHE score of 30 patients. We had no missing values regarding mortality. Seventy-three patients (24%) were classified as having sepsis using the “old” 2003 criteria, compared with 123 patients (41%) using the “new” 2016 criteria. Three patients (1%) were classified as having sepsis with the 2003 criteria and not with the 2016 criteria.

MPO levels higher than 91 ng/mL—the cutoff between the fourth and fifth quintile—were related to higher mortality, higher APACHE IV scores, higher SOFA scores, and higher leukocyte levels (Table 1). The three patients with missing values for MPO were excluded from further analysis. They did not have sepsis during their ICU stay, and one of the three died within 30 days. There was no relation between MPO and the development of infection on different infection sites (Table 1).

**Table 1:** Descriptive characteristics, medians (IQR) or *N* (%)

Characteristics	Overall
Number of patients	313
Gender, male	193 (62%)
Age, years	61 (24)
Diagnosis at admission	
Trauma	23 (7.3%)
Infection (suspicion of)	79 (25%)
Post-surgery	53 (17%)
Other	155 (50%)
Severity of illness (at admission)	
Mechanical ventilation	202 (66%)
APACHE IV score	75 (40)
SOFA score	6 (6)
ICU stay, days	9 (10)
Sepsis at admission to ICU	123 (39%)
Mortality 30 days	77 (25%)
Site of infection sepsis patients^a^ (*N* = 123)	
Skin/wound	12 (10%)
Pneumonia	58 (47%)
Line sepsis	11 (9%)
Abdominal	12 (10%)
Urinary tract	3 (2.4%)
Other/unknown	41 (33%)
Proven infection in sepsis patients (*N* = 123)	62 (50%)
CRP, mg/L	216 (189)
Leukocytes max, × 10^9^/L	16 (10)

*IQR* indicates interquartile range

^a^14% mixed infections

### MPO and infection

**Fig. 1:**
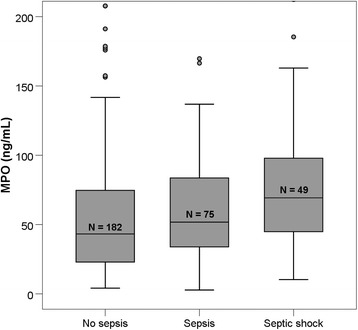
Myeloperoxidase (MPO) levels in different states of inflammation. Septic shock has higher MPO levels compared with sepsis, *P* = 0.04. Patients with sepsis (sepsis + septic shock) have higher MPO levels compared with patients without sepsis, *P* = 0.002

**Fig. 2:**
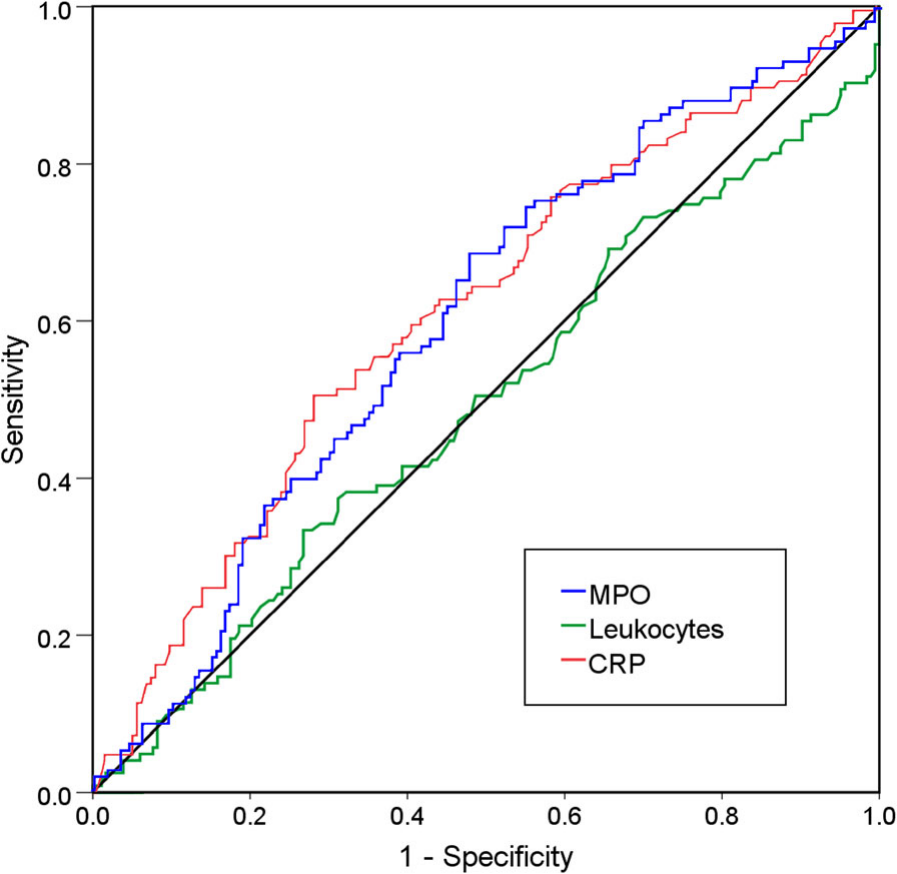
ROC curves of MPO, CRP, and leukocytes in relation to sepsis. MPO myeloperoxidase, CRP C-reactive protein. MPO and CRP are both related to sepsis with an AUC 0.603 (CI 0.539–0.668) and AUC 0.636 (CI 0.573–0.699). Leukocytes have no discriminative value

The MPO levels in patients with sepsis were significantly higher compared with patients without sepsis (60 ng/mL [IQR 36–88 ng/mL] versus 43 ng/mL [IQR 23–75 ng/mL], *P* = 0.002). MPO levels were significantly raised in patients with septic shock compared with patients without septic shock (69 ng/mL [IQR 41–98] versus 48 ng/mL [IQR 26–78], *P* < 0.001, area under the curve (AUC) 0.647 [CI 0.559–0.736)]) (Fig. 1, myeloperoxidase (MPO) levels in different states of inflammation). CRP and MPO distinguished sepsis from non-infectious SIRS similarly (MPO: AUC 0.603 [95% confidence interval (CI) 0.538–0.668] versus CRP: AUC 0.636 [CI 0.572–0.700]). Leukocytes did not distinguish sepsis from non-infectious SIRS in our population (AUC 0.493 [CI 0.427–0.559]). ROC-curves are shown in Fig. 2.

### MPO, mortality and APACHE IV

**Fig. 3:**
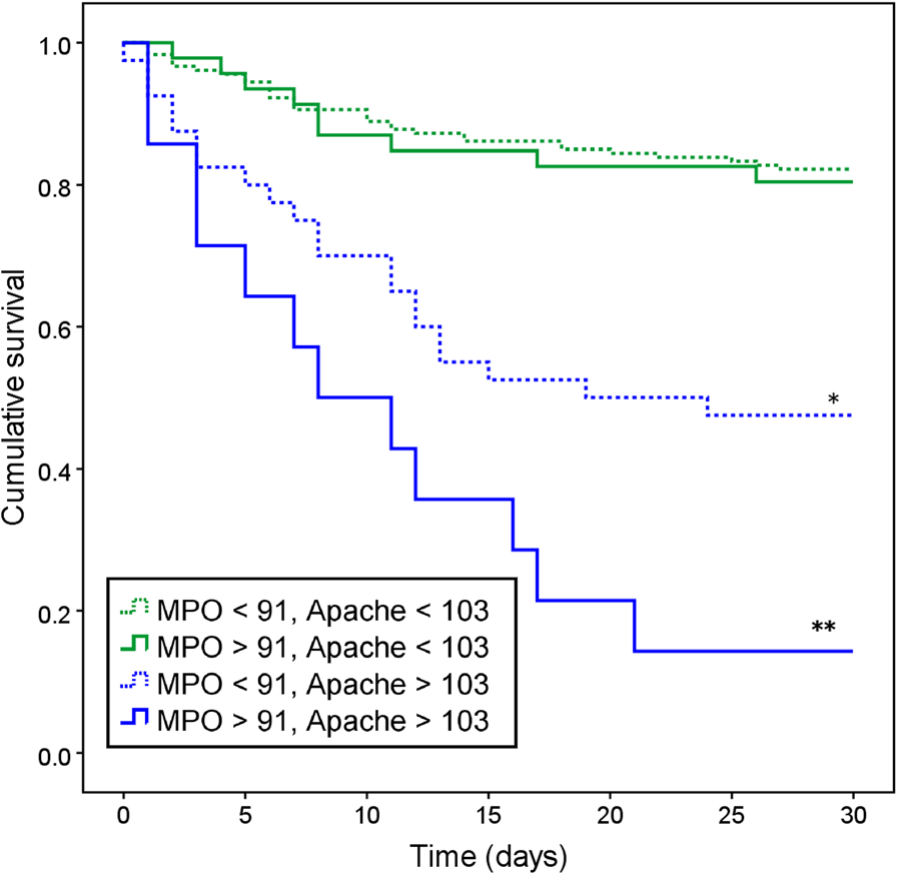
Kaplan Meier 30-day survival curve. MPO myeloperoxidase, APACHE Acute Physiology And Chronic Health Evaluation. Single asterisk indicates significant difference with upper lines (*P* > 0.001), and double asterisks indicate significant difference with third line (*P* = 0.028)

The maximum level of MPO of the first 48 h was correlated to 30-day mortality (*P* = 0.032) and to the APACHE IV score (*P* < .001). When we compared the upper 20% MPO levels with the lower 80% MPO levels, we found a mortality hazard ratio of 1.8 [CI 1.07–2.88, *P* = 0.02]. Our data show that MPO could differentiate between survival and non-survival when added to the APACHE IV score. In patients with a lower APACHE score (< 103, cutoff between fourth and fifth quintile), MPO did not differentiate and the general survival rate was 82%. In patients with a high APACHE score (> 103), MPO did differentiate between survival and non-survival. With a high APACHE score and a lower MPO level, survival was 48.5%; with both a high APACHE score and a high MPO level, the survival dropped to 14.3% (*P* = 0.028, Fig. 3, Kaplan Meier 30-day survival curve, and Table 2). The sensitivity of MPO with a cutoff at the fifth quintile in patients with an APACHE IV score > 103 was 90.5%, with a specificity of 36.4%. In contrast to MPO, CRP is unrelated to mortality in both the general (*P* = 0.47) group and in the groups with low and high APACHE scores (*P* = 0.51 and *P* = 0.46).

**Table 2:** Mortality risk and hazard ratios

	*N* (%)	Deaths	Mortality	Hazard ratio	95% CI
MPO < 91, APACHE < 103	180 (64)	32	18%	1.0	n/a
MPO > 91, APACHE < 103	46 (16)	9	20%	1.1	0.5–2.3
MPO < 91, APACHE > 103	40 (14)	21	53%	3.8	2.2–6.6
MPO > 91, APACHE > 103	14 (5)	12	86%	8.1	4.1–16
Total	280 (100)	74	26%		

## Discussion

In our cohort study of ICU patients, plasma MPO levels differed between SIRS and sepsis patients: plasma levels of MPO in patients with sepsis were higher than in patients with SIRS without (suspicion of) infection. MPO could also differentiate between patients with sepsis and patients with septic shock. Higher MPO levels were associated with a higher mortality. In patients with a high APACHE IV score, MPO was able to discern between survivors and non-survivors.

The relationship between sepsis and elevated MPO levels as found in our study can be explained by the leakage of MPO into the plasma when neutrophils phagocyte bacteria [12]. Prior to our study, a limited amount of studies investigated the discriminative value of MPO between non-infectious SIRS and sepsis. In our patient cohort, MPO could differentiate between SIRS without infection and sepsis. In contrast, a previously published study by Kothari et al. [13] showed no difference in the MPO levels between non-infectious SIRS and sepsis, and their data showed lower MPO levels in patients with septic shock. A difference between this study and ours is the use of the “old” sepsis criteria, the age of their patients (38 years versus 60 years—since they excluded patients older than 80 years) and a higher incidence of organ failure (mean SOFA scores 12 versus 7). Another study, done by Cha et al. [14], showed a relation between sepsis and MPO. Both aforementioned studies used a different approach to measuring MPO compared with our study. The study of Kothari et al. used o-dianisidine to measure MPO activity; the study of Cha et al. used the mean myeloperoxidase index (MPXI), which measures the MPO intracellular. MPO activity can vary even if levels of MPO are similar both due to genetic differences and the ability of MPO to bind to negatively charged surfaces (like albumin) [15, 16]. The comparison between the different methods to measure MPO within infected patients is unknown. We show that serum MPO levels, independent of their activity, are a marker of inflammation. However, it would be interesting to test both MPO activity and intra- and extracellular MPO levels concurrently during infection and/or sepsis.

We compared the discriminative value between sepsis and non-infectious SIRS of MPO with CRP and leukocytes. We show that MPO had a similar discriminative value to CRP and that leukocytes did not differentiate between non-infectious SIRS and sepsis. An advantage of MPO to CRP could be the predictive value for mortality; however, the additional value in clinical practice is debatable. Procalcitonin, an indicator of severity of illness, is used increasingly in the ICU, and for further biomarker studies, we would advise to compare the biomarker with procalcitonin (next to CRP/leukocyte count) [17].

In general, the relation between MPO and mortality could be explained by two mechanisms. Firstly, MPO and the products formed by MPO could directly lead to extracellular collateral damage and consequently be the direct cause of higher mortality. Secondly, MPO could be a marker of inflammation. Inflammation has been shown to lead to multiple organ failure and therefore to higher mortality [18]. To our knowledge, this is the only study researching the value of MPO as a biomarker predicting mortality in the ICU. Studies performed in patients with myocardial infarction showed that plasma MPO levels are related to mortality as seen in our study [19, 20]. The authors also found MPO to be related to coronary artery disease and thus explained the relation of MPO with mortality.

Our data show that MPO is of additional value to the APACHE IV score in the 20% most critically ill patients. This could be explained by the poor calibration and the general underestimation of mortality risk associated with the APACHE IV model [21, 22]. The addition of a biomarker, which better discriminates between surviving and dying patients, might correct the underestimation of the APACHE IV model. This partially explains why MPO levels especially differentiate in patients with the 20% highest APACHE IV score.

Our study is one of few studies assessing plasma MPO as a discriminative biomarker and the only study assessing plasma MPO as a prognostic biomarker in the ICU. A limitation of our study is that we used the SIRS criteria—as published in 2003—as inclusion criteria. Those SIRS criteria have been criticized because they lack sensitivity [1]. For future studies, we recommend abandoning the SIRS criteria and instead including all ICU patients (with an estimated stay of more than 24 h). MPO assays are already widely available; therefore, it should be fairly easy to implement measurement of plasma MPO in ICU work-up. Since our study is performed in a fairly large cohort in a general ICU and had few missing values, our results should be generalizable to other ICUs.

## Conclusions

In conclusion, we show that plasma levels of MPO correlate with severity of disease in SIRS patients in the ICU and discriminate between sterile inflammation and sepsis. Secondly, we show that MPO has a prognostic value next to the APACHE IV score. However, the additional value to current biomarkers should be further assessed.

## Abbreviations

APACHE: Acute Physiology and Chronic Health Evaluation
CI: 95% Confidence interval
CRP: C-reactive protein
ICU: Intensive care unit
IQR: Interquartile range
MPO: Myeloperoxidase
SIRS: Systemic inflammatory response syndrome
SOFA: Sequential Organ Failure Assessment
